# Feasibility and acceptability of an online multicomponent very low-carbohydrate intervention in young adult women with obesity: a pilot study

**DOI:** 10.1186/s40814-024-01525-0

**Published:** 2024-07-29

**Authors:** Laura R. Saslow, Alison O’Brien, Kaitlyn Raymond, Hovig Bayandorian, Deanna Marriott, Judith T. Moskowitz, Jennifer Daubenmier, Dave Bridges, Cody M. Cousineau, Dina H. Griauzde

**Affiliations:** 1 Department of Health Behavior and Biological Sciences, School of Nursing, University of Michigan, Ann Arbor, MI USA; 2 Berkeley, CA USA; 3 Department of Medical Social Sciences, Feinberg School of Medicine, Northwestern University, Chicago, IL USA; 4 Institute of Holistic Health Studies, San Francisco State University, San Francisco, CA USA; 5 Department of Nutritional Sciences, University of Michigan School of Public Health, Ann Arbor, USA; 6 VA Ann Arbor Healthcare System, Ann Arbor, MI USA; 7 University of Michigan Medical School, Ann Arbor, MI USA

**Keywords:** Weight loss, Young adults, Online intervention, Dietary, Ketogenic

## Abstract

**Background:**

Approximately one-third of US young adults (18–25 years) have obesity, and there are calls to help young adults lose weight to prevent weight-related chronic conditions. This pilot trial tested the feasibility and acceptability of a very low-carbohydrate (VLC) eating pattern, with supportive positive affect and mindful eating skills, for weight management among young females with obesity.

**Methods:**

In a single-arm trial, women (*N* = 17), aged 19–23, with obesity participated in a 4-month diet and lifestyle intervention. Participants were taught how to follow a VLC eating pattern with the help of a coach and 16 weekly web-based sessions. We assessed feasibility and acceptability through session participation, outcome collection, intervention satisfaction, and adverse events.

**Results:**

Seventeen participants enrolled and 14 (82%) reported body weight at 4 months. Fifteen participants (94% of those beginning the intervention) viewed at least one session, and 8/15 (53%) of these participants were active in the intervention, viewing at least half of the sessions. Among the nine participants who provided 4-month self-report information, intervention satisfaction was high (mean 5.89/7, 95% CI 4.59 to 7.19). Among participants with a 4-month body weight, 7/14 (50%) lost ≥ 5% of their body weight, and of those who were also active in the intervention, 6/7 (86%) lost ≥ 5% of their body weight. There were no serious adverse events.

**Conclusions:**

The results of this pilot study suggest that a VLC eating pattern may be a feasible and acceptable approach for weight loss in some young women with obesity.

**Trial registration:**

This trial was registered with ClinicalTrials.gov on August 18, 2021. The trial number is NCT05010083.

**Supplementary Information:**

The online version contains supplementary material available at 10.1186/s40814-024-01525-0.

## Key messages regarding feasibility

- *What uncertainties existed regarding the feasibility?* The feasibility of an online very low-carbohydrate diet program for young adults is currently unknown.
- *What are the key feasibility findings?* Seventeen participants enrolled and 14 (82%) reported body weight at 4 months. Fifteen participants (94% of those beginning the intervention) viewed at least one session, and 8/15 (53%) of these participants were active in the intervention, viewing at least half of the sessions.
- *What are the implications of the feasibility findings for the design of the main study?* Among the nine participants who provided 4-month self-report information, intervention satisfaction was high (mean 5.89/7, 95% CI 4.59 to 7.19). Future trials should compare this approach to dietary patterns in a larger, more diverse population.

## Introduction

Approximately one-third of US young adults (18–25 years) have obesity, defined as a body mass index (BMI) of ≥ 30 kg/m^2^ [1]. Those who are overweight in young adulthood are more likely to develop hypertension, type 2 diabetes, cardiovascular disease, and obesity-related cancer later in life [2–4]. Thus, there are calls to prevent and treat obesity and excess weight gain among young adults [5, 6] Young women, in particular, are especially vulnerable to excess weight gain [7].

A very low-carbohydrate (VLC or ketogenic) eating pattern is one potentially effective weight control approach, as the reduction of carbohydrates can lower insulin secretion and promote the conversion of fatty acids to ketone bodies for energy consumption, typically leading to weight loss, reductions in hunger, better glucose control, and improvements in cholesterol and lipids [8]. While a VLC eating pattern has been successfully used to support weight loss among adult populations, little is known about the feasibility, acceptability, or weight loss effectiveness of this dietary approach among young adults. Young adults are more challenging to recruit and retain in weight loss interventions than older adults. For example, in a study of more than 41,000 individuals in Diabetes Prevention Programs, researchers found that age was positively correlated with retention in the intervention, with 18–29-year-old participants having the lowest retention rate; only 46% of young adults who attended at least the first class were retained at 18 weeks [9]. Moreover, young adults often lose less weight, on average, than older adults in diet and lifestyle interventions [10–13] Dietary adherence can also be challenging for young adults. For example, no participants assigned to a VLC eating pattern in a recent trial of young adults with type 1 diabetes successfully followed the dietary intervention [14]. Moreover, young adults are challenging to retain in diet and lifestyle programs.

We therefore aimed to pilot test a VLC dietary intervention among young adults with obesity. We included two types of psychological skills: (a) positive affect skills, which aim to increase the frequency that participants experience positive emotions, to improve intervention adherence and satisfaction [15, 16], and (b) mindful eating, to help participants cope with emotional and hedonic-driven eating, drivers of weight gain in young adults and adults more broadly [17–19]. The intervention also incorporated all of the key strategies recently described in a review of online health weight management interventions for young adults: strategies to support self-regulation, including goal setting and self-monitoring, personalized feedback and support from a coach, social support, reminders, and booster messages [20].

In this trial of young adults with obesity, we examined intervention feasibility and acceptability and physical and patient-reported outcomes.

## Materials and methods

### Study design

This was a single-arm pilot trial of an online, VLC dietary intervention.

#### Participants and setting

Inclusion criteria included age 18–25 years and BMI ≥ 30 kg/m^2^. Our target sample size was 30 participants. Exclusion criteria included having a self-reported eating disorder, following a vegan or vegetarian eating pattern, already following a VLC eating pattern, being pregnant or breastfeeding, or having a health condition not deemed safe for participation. The intervention was delivered online.

#### Recruitment and screening

Potential participants were identified using advertisements on social media. Online ads directed participants to a web page with a description of the trial, study contact information, and a link to an online screening survey (Qualtrics.com). The screening survey included questions used to assess eligibility. If individuals appeared eligible based on their survey responses, they were sent a video describing the goals, procedures, and potential risks and benefits of participating in the trial. Those who remained interested provided written informed consent. Baseline measures included an online survey, dietary recall, body weight measurement, and at-home HbA1c test. Those who completed all baseline measures were enrolled in the trial.

#### Intervention

The online intervention included 16 weekly e-mails containing an educational video, a survey to assess dietary adherence and self-reported physical and mental health, and handouts. Participants also received text messages and e-mail-based coaching. At baseline, participants received a body weight scale (BodyTrace) and urine ketone strips (which can detect urinary ketones in a noninvasive way, but preliminary research show that their accuracy may be lower than measurements with blood ketones) [21]. Participants were also sent VLC cookbooks by mail three times throughout the intervention [22–24]. We instructed participants to follow a VLC eating pattern, consisting of 20–35 net (non-fiber) grams of carbohydrate per day. The intervention encouraged participants to engage in at least 150 min of moderate intensity physical activity per week, to get enough sleep to feel well rested, to regularly monitor their dietary intake using MyFitnessPal, to self-weigh at least once a week, and to apply positive affect [25] and mindful eating skills [17, 26]. The intervention also provided personalized feedback and support from the coach, social support (by teaching relevant skills for increasing social support with their friends and family, plus encouragement to use online VLC-focused postings and resources for social support), and reminders and booster messages (from the main intervention content and the accompanying text messages).

#### Data collection

At baseline, participants were asked to self-weigh using the scale provided by the study, measure their hemoglobin A1c (HbA1c) using a mail-away kit (DTI Laboratories), complete an online survey (Qualtrics.com), and complete two 24-h dietary recalls over the phone. Survey measures included the validated Patient Health Questionnaire-4 (PHQ-4) [27] to assess symptoms of depression (two items) and anxiety (two items). At 4 months, the end of the intervention, these measures were repeated, with additional questions concerning intervention feasibility and acceptability. Participants were also asked about their dietary adherence and experiences each week throughout the intervention via a brief self-report survey.

### Outcome measures

#### Primary intervention feasibility

##### Trial and intervention retention

We defined trial retention as the percent of participants who completed the post-intervention outcomes divided by the total number of trial participants enrolled. We assessed intervention retention based on intervention participation. The National Diabetes Prevention Program, led by the Centers for Disease Control and Prevention, is a nationwide intervention aimed at reducing the risk of developing type 2 diabetes through weight loss and increased physical activity. According to their standards, one benchmark for success of the intervention is that 50% of individuals who attend the first session of the intervention are retained in the intervention by the fourth month [28]. We explored an adapted benchmark of success for our intervention, whether at least 50% of individuals who view at least one session of the intervention actively participate in the intervention, defined here as viewing at least 8/16 or 50% of the program sessions.

##### Dietary adherence

Each week, starting in week 3, participants were asked whether their urinary ketone strips showed increased ketone levels. We also asked whether, based on self-assessment, they were following a VLC eating pattern rated from 1 = “not at all” to 7 = “very much so.” In addition, at baseline and 4 months, participants completed two 24-h dietary recalls over the phone. We defined VLC dietary adherence as an average 4-month net carbohydrates of 60 g/day or lower, based on these two dietary recalls.

#### Primary intervention acceptability

##### General intervention satisfaction

At month 4, we asked participants, “How would you rate your overall satisfaction with the program?” Response options ranged from 1 = “not at all satisfied” to 7 = “very satisfied.” We set as a feasibility threshold that on average, participants would rate this above the scale’s midpoint of 4. To assess potential acceptability, we also asked participants to answer the following: “How long can you see yourself following your assigned diet?”.

##### Intervention skills satisfaction

At month 4, participants rated their satisfaction with both the positive affect skills and mindfulness skills included in the intervention. Response options ranged from 1 = “don’t include them, they were not helpful” to 7 = “you must include them, they were very helpful.”

##### Qualitative feedback

As part of the weekly sessions, we asked participants a variety of open-ended questions throughout the intervention, such as “What are your favorite low-carb snacks?” and “When you need a quick low-carb meal, what do you tend to make or grab?” At 4 months, using open-ended questions, we asked participants about their experiences with and recommendations for the intervention overall. We summarized these responses, focusing on their suggestions for improving the intervention, what they liked about the intervention, and what factors they thought prevented or supported their adherence to the program.

#### Secondary patient-centered objectives

##### Body weight changes

Participants self-weighed throughout the trial, using the provided scale. Weight data were sent automatically to trained study staff through the scale’s connection to a cellular network. Mean change in weight and BMI (using baseline self-reported height) was calculated at 4 months compared to baseline. Mean percent weight loss was defined as follows: (weight at 4 months − baseline weight)/(baseline weight) × 100.

##### Achievement of ≥ 5% body weight loss

We calculated the percent of participants achieving ≥ 5% weight loss, a clinically significant threshold [29]. According to the standards of the National Diabetes Prevention Program, a benchmark for success of the intervention is that 60% of active participants (who view at least 8/16 or 50% of sessions in the first 6 months) lose at least 5% of their body weight within 12 months of beginning the trial [28]. We explored an adaptation of these requirements for our trial. We considered 60% of active participants (participants who viewed at least 8/16 or 50% sessions in the 4 months of the intervention) losing 5% of their body weight by 4 months to be a sign of preliminary efficacy for the outcome of the trial.

##### HbA1c changes

HbA1c was measured at baseline and month 4 using home-based, mail-away kits (DTI Laboratories). Mean change in HbA1c was calculated at 4 months compared to baseline. The laboratory was masked to study design.

##### Self-rated change in health

At month 4, we asked participants to rate how their health changed over the intervention with the question, “How much do you think your health has changed as a result of participating in this program?” Response options ranged from 1 = “very much worse” to 7 = “very much better.”

##### Changes in psychological factors

We assessed changes from baseline to 4 months for symptoms of anxiety and depression, measured with Patient Health Questionnaire-4 (PHQ-4), [27] positive affect with the Scale of Positive and Negative Experience (SPANE), [30] mindful eating with the reliance on Hunger and Satiety Cues subscale of the Intuitive Eating Scale-2, [31] and stress-based eating with the Palatable Eating Motives Scale (PEMS) coping subscale [32].

### Analysis

We completed an intention-to-treat analysis with all available data, with no imputation for missing data. Means and 95% confidence intervals were calculated for continuous variables.

## Results

### Sample characteristics

Of the 221 individuals who expressed interest in study participation and were screened for study eligibility, 204 did not meet inclusion criteria (BMI too low: *n* = 165; age out of range: *n* = 14; self-reported eating disorder: *n* = 8; vegan or vegetarian: *n* = 4; already following a VLC eating pattern: *n* = 3; pregnant or breastfeeding: *n* = 1; a health condition not deemed safe for participation: *n* = 1), 7 were eligible but declined to participate, and 18 did not respond to follow-up study team questions. Thus, among those screened, 24 (11%) were eligible for study participation. Among eligible individuals, 17 (71%) enrolled in the trial. One participant withdrew for mental health reasons before beginning the intervention but after study enrollment. We did not reach our target sample size of 30 participants within the time and budget allowed for the study conduct.

At baseline, participants were 19–23 years old (mean 20.12 years, SD 0.93), with a mean BMI of 39.74 kg/m^2^ (SD 7.88 kg/m^2^, ranging from 27.49 to 54.98). All were female, and most were white (14/17 or 82%, with 1 being Asian, 2 being African-American, 1 being American Indian or Alaska Native) and non-Hispanic (13/17 or 76%). The mean PHQ-4 score was 4.41, representing mild average baseline levels of depression and anxiety. Additional characteristics and baseline measures are shown in Table 1.

**Table 1:** Participant characteristics

Characteristics	*N* = 17
Age, years; M (SD)	20.12 (SD 0.93)
Sex: female, %	100
Race: White, %	82
Weight, kg; M (95% CI)	104.67 (94.82 to 114.51)
BMI, kg/m^2^; M (95% CI)	39.74 (35.68 to 43.79)
HbA1c, mmol/mol; M (95% CI)	32.18 (30.51 to 33.84)
Patient Health Questionnaire-4; M (95% CI)	4.41 (2.51 to 6.31)
Positive affect; M (95% CI)	20.29 (18.3 to 22.28)
Mindful eating; M (95% CI)	3.05 (2.56 to 3.54)
Stress-based eating; M (95% CI)	2.75 (2.15 to 3.35)
Energy, kilocalories; M (95% CI)	1529.61 (1318.06 to 1741.15)
Carbohydrates, g; M (95% CI)	171.14 (144.40 to 197.87)
Fiber, g; M (95% CI)	12.76 (9.29 to 16.24)
Net (non-fiber) carbohydrates, g; M (95% CI)	158.10 (133.39 to 182.82)
Total fat, g; M (95% CI)	64.01 (49.60 to 78.42)
Monounsaturated fat, g; M (95% CI)	18.64 (13.65 to 23.63)
Polyunsaturated fat, g; M (95% CI)	10.21 (7.17 to 13.25)
Saturated fat, g; M (95% CI)	22.88 (17.46 to 28.31)
Protein, g; M (95% CI)	70.39 (58.71 to 82.08)

#### Primary intervention feasibility

##### Trial and intervention retention

Of the 17 participants who enrolled in the study, 82% (*n* = 14) provided their post-intervention body weight, 65% (*n* = 11) took part in the post-intervention 24-h dietary recalls, 53% (*n* = 9) completed the post-intervention survey, and 47% (*n* = 8) completed the post-intervention HbA1c test. Fifteen participants viewed at least one session of the intervention, and 8/15 (53%) attended at least half of the sessions, reaching our exploratory goal of 50%.

##### Dietary adherence

Across all participants, the mean of the weekly self-reported dietary adherence rating was 3.84 (95% CI 2.65 to 5.02), and the mean number of weeks that participants reported a positive urine ketone strip was 4.53 (95% CI 1.70 to 7.01). Eleven participants completed the pre- and post-intervention dietary recalls. Changes in nutrient intake are shown in Table 2. In Table 3, we show results for the 6 participants (6/11 or 54.5% of people with dietary recalls) who were adherent to the eating pattern. Across all participants at 4 months, participants reported a reduction in net carbohydrate intake to 70 g/day. For participants who were adherent to the VLC diet, at 4 months, they reported a reduction in net carbohydrate intake to 33 g/day. For the participants adherent to the eating pattern according to the 24-h dietary recall, the mean of the weekly self-reported dietary adherence rating was 5.78 (95% CI 4.80 to 6.77), and the number of weeks that participants reported a positive urine ketone strip was 7.83 (95% CI 1.96 to 13.71).

**Table 2:** Outcomes for all participants with outcome data

Outcomes	Baseline (mean, 95% CI)	Post (mean, 95% CI)	Percent change	Change (mean, 95% CI)
Weight, kg, *n* = 14	105.80 (93.85 to 117.74)	102.66 (92.53 to 112.79)	− 3.46	− 3.14 (− 6.94 to 0.67)
BMI, kg/m^2^, *n* = 14	40.33 (35.64 to 45.02)	39.01 (34.85 to 43.17)	− 3.69	− 1.32 (− 2.74 to 0.09)
HbA1c, mmol/mol, *n* = 8	31.5 (28.83 to 34.17)	32.5 (31.27 to 33.73)	2.96	1.00 (− 1.14 to 3.14)
Patient Health Questionnaire-4, *n* = 9	2.67 0.88 to 4.46)	2.33 (0.19 to 4.47)	− 26.67	− 0.33 (− 4 to 3.33)
Positive affect, *n* = 9	22 (20.89 to 23.11)	24.22 (23.75 to 24.69)	11.25	2.22 (− 1.1 to 5.55)
Mindful eating, *n* = 9	3.28 (0.05 to 6.51)	3.65 (1.18 to 6.12)	22.66	0.37 (− 0.45 to 1.19)
Stress-based eating, *n* = 9	2.39 (1.81 to 2.97)	2.17 (1.48 to 2.86)	− 5.37	− 0.22 (− 1.41 to 0.96)
Energy, kilocalories, *n* = 11	1580.43 (1579.44 to 1581.42)	1247.05 (1246.29 to 1247.81)	− 19.64	− 333.39 (− 608.62 to − 58.16)
Carbohydrates, g, *n* = 11	176.74 (− 58.32 to 411.8)	89.72 (− 118.42 to 297.86)	− 49.42	− 87.02 (− 123.67 to − 50.36)
Fiber, g, *n* = 11	13.34 (− 20.02 to 46.7)	18.32 (− 8.17 to 44.81)	51.69	4.98 (− 3.75 to 13.71)
Net (non-fiber) carbohydrates, g, *n* = 11	163.05 (155.51 to 170.59)	70.01 (66.13 to 73.89)	− 57.52	− 93.03 (− 129.22 to − 56.84)
Total fat, g, *n* = 11	64.95 (31.95 to 97.95)	66 (41.6 to 90.4)	12.95	1.04 (− 17.95 to 20.04)
Monounsaturated fat, g, *n* = 11	18.88 (4.32 to 33.44)	15.63 (0.29 to 30.97)	− 12.35	− 3.25 (− 9.76 to 3.26)
Polyunsaturated fat, g, *n* = 11	9.87 (4.19 to 15.55)	6.54 (1.36 to 11.72)	− 22.98	− 3.34 (− 6.61 to − 0.06)
Saturated fat, g, *n* = 11	24.57 (22.61 to 26.53)	27.6 (24.06 to 31.14)	32.79	3.03 (− 5.8 to 11.85)
Protein, g, *n* = 11	76.13 (69.37 to 82.89)	81.74 (75.24 to 88.24)	7.60	5.61 (− 11.05 to 22.27)

**Table 3:** Outcomes for participants who were adherent to the eating pattern (*N* = 6)

Outcomes	Baseline (mean, 95% CI)	Post (mean, 95% CI)	Percent change	Change (mean, 95% CI)
Weight, kg	104.81 (85.94 to 123.68)	96.80 (74.70 to 118.90)	− 8.18	− 8.01 (− 14.06 to − 1.96)
BMI, kg/m^2^	39.56 (31.48 to 47.64)	36.48 (27.56 to 45.4)	− 8.18	− 3.08 (− 5.52 to − 0.65)
HbA1c, mmol/mol	31.33 (29.9 to 32.77)	33.00 (30.85 to 35.15)	5.11	1.6 (0.49 to 2.71)
Patient Health Questionnaire-4	3.00 (− 0.38 to 6.38)	1.83 (− 1.45 to 5.11)	− 65.00	− 1.17 (− 6.81 to 4.48)
Positive affect	22.67 (18.93 to 26.4)	25 (19.57 to 30.43)	11.89	2.33 (− 3.25 to 7.91)
Mindful eating	3.03 (1.94 to 4.12)	3.83 (3.29 to 4.38)	41.85	0.81 (− 0.28 to 1.89)
Stress-based eating	2.46 (1.11 to 3.81)	1.46 (0.98 to 1.94)	− 34.19	− 1.00 (− 2.16 to 0.16)
Energy, kilocalories	1503.08 (1140.18 to 1865.98)	1068.94 (822.28 to 1315.6)	− 28.20	− 434.14 (− 626.56 to − 241.72)
Carbohydrates, g	156.7 (129.52 to 183.88)	53.63 (26.96 to 80.29)	− 66.19	− 1 03.07 (− 129.43 to − 76.72)
Fiber, g	13.4 (5.61 to 21.19)	19.14 (1.75 to 36.53)	43.13	5.74 (− 10.09 to 21.57)
Net (non-fiber) carbohydrates, g	143.08 (119.16 to 167)	32.37 (13.72 to 51.02)	− 76.98	− 110.72 (− 139.41 to − 82.02)
Total fat, g	69.24 (40.88 to 97.6)	61.43 (34.96 to 87.9)	− 6.77	− 7.81 (− 25.4 to 9.78)
Monounsaturated fat, g	16.92 (8.86 to 24.99)	14.16 (5.29 to 23.03)	− 17.74	− 2.77 (− 8.75 to 3.22)
Polyunsaturated fat, g	7.58 (4.23 to 10.92)	6.03 (2.29 to 9.76)	− 20.37	− 1.55 (− 3.69 to 0.59)
Saturated fat, g	29.35 (17.84 to 40.86)	27.03 (16.2 to 37.87)	− 3.22	− 2.32 (− 11.37 to 6.73)
Protein, g	68.38 (53.56 to 83.21)	84.57 (63.53 to 105.61)	24.87	16.18 (0.03 to 32.34)

#### Primary intervention acceptability

##### General intervention satisfaction

Of the 9 participants who provided 4-month self-report information, intervention satisfaction was high (mean 5.89, 95% CI 4.59 to 7.19), with 8/9 (89%) rating the intervention a 5 out of 7 or higher. This reached our feasibility threshold that on average, participants would rate this above the scale’s midpoint of 4. Just less than half, 4/9 (44%) reported that they would stop the assigned eating pattern as soon as the study was over, with 4/9 (44%) reporting that they intended to continue the eating pattern for at least another few months and 1/9 (11%) stating that they did not plan to ever stop following a VLC eating pattern.

##### Intervention’s skills satisfaction

The nine participants who provided 4-month self-report information rated their satisfaction with the positive affect skills (mean 5.89, 95% CI 4.99 to 6.79), with 4/9 or 44% rating them the top score. Participants rated their satisfaction with the mindfulness skills (mean 5.89, 95% CI 5.08 to 6.70), with 3/9 or 33% rating them the top score.

##### Qualitative feedback

Participants liked most aspects of the intervention, including the weekly videos, the body weight scale, the recipes, and access to the supportive personal coach. Several participants noted feeling more energetic. Some participants suggested adding phone or video chats with the dietary coach, adding an online discussion board or other way to interact with other participants in the intervention, sending more cooking videos, adding more examples about how to understand the dietary content of what they were eating, and making dietary tracking mandatory.

Participants mentioned factors that supported their adherence to and motivation for the intervention, including accountability by being in the intervention, preparing their meals ahead of time, feeling motivated by the weight loss success of others, seeing their own initial weight loss success, and support from the intervention coach. They noted barriers that reduced their adherence to and motivation for the intervention, including stress, lack of time, budgetary constraints, feeling that the VLC eating pattern was too restrictive, being around family members not eating a VLC eating pattern, and eating out of boredom.

#### Secondary patient-centered objectives

##### Adverse events

No serious adverse events were reported, but adverse events included a manic episode (for one participant with bipolar disorder, who only viewed three sessions) and hair loss that stopped after the participant increased her carbohydrates (for one participant, who also only viewed three sessions).

##### Body weight changes

Across all participants with a post-intervention body weight, participants lost about 3.5% of body weight (Table 2). However, for the participants who were adherent to the eating pattern, participants lost about 8.2% of body weight (Table 3).

##### Achievement of ≥ 5% body weight loss

Among participants with a 4-month body weight, 7/14 (50%) of participants lost at least 5% of their body weight. Of those who were adherent to the eating pattern, 5/6 (83%) lost at least 5% of their body weight. Of those active in the intervention with a 4-month body weight, 6 out of 7 (86%) lost at least 5% of their body weight, higher than our goal of 60%, suggesting preliminary efficacy of the trial.

##### HbA1c changes

Across all participants, HbA1c stayed in the non-clinically significant range (Table 3).

##### Self-rated change in health

One participant rated her health as having gotten a little worse, 1 reported that her health had not changed, 2 reported their health got a little better, 4 reported that their health got much better, and 1 reported that their health was very much better.

##### Changes in psychological factors

Across participants and for participants who were adherent to the eating pattern, changes were in the expected direction, with positive affect and mindful eating increasing and depressive symptoms and stress-based eating decreasing.

## Discussion

The results of this pilot study of a single-arm, online, VLC dietary intervention suggest that a VLC eating pattern may be feasible and acceptable for weight management among young adult women with obesity.

In terms of acceptability, overall, participants were satisfied with the intervention, including the positive affect and mindful eating components. However, they did suggest some possible changes to the intervention, such as phone calls with the coach and interaction with other participants. In other future work, therefore, we could consider testing this addition to the intervention. There is research to suggest that added support might be of help. For example, a recent intervention optimization trial to increase physical activity in breast cancer survivors demonstrated a greater increase in physical activity among sedentary participants assigned to receive six biweekly 10- to 15-min coaching calls (versus no calls) [33].

In this trial, 6 of 7 (86%) of active participants lost at least 5% of their body weight, higher than our goal of at least 60%. This result might be considered a clinically meaningful preliminary finding, considering typical weight loss results in this age group. For example, in a systematic review of weight-loss interventions for young adults, only 4 out of 12 trials that focused on nutrition reported statistically significant weight loss [34]. Similarly, in a study of more than 14,500 adults who used on online app for weight loss, the younger the participants, the less the weight loss [13].

As HbA1c levels of 5.7% and higher are considered to be in the prediabetic range, HbA1c did not change a clinically meaningful amount in this trial. Of note, in a previous trial, HbA1c had low sensitivity and specificity in children and young adults, so HbA1c may not be an appropriate marker of glycemic control in this population [35].

Although no serious adverse events were reported, one participant with bipolar disorder experienced a manic episode. Preliminary research suggests that a VLC eating pattern to induce nutritional ketosis can be a treatment for bipolar disorder, but medication management and oversight should occur [36]. One participant reported hair loss, which can be a transient feature of all weight loss regardless of the approach [37]. Several participants noted feeling more energetic, a finding that has been reported previously in VLC eating pattern studies [38, 39].

### Limitations

We were able to successfully recruit participants for this online trial. However, our recruitment strategy identified a high proportion of ineligible individuals, suggesting that social media may not be an efficient strategy for recruiting young adults for this intervention. Future efforts to recruit this population may consider engaging individuals’ physicians or using medical records to identify eligible individuals. Moreover, this trial’s generalizability is limited, as most participants were white, non-Hispanic women, which is typical of weight-loss trials of young adults [40]. Future recruitment efforts should focus on recruiting a more diverse population. In addition, the trial was conducted entirely online, with no in-person visits, during the COVID-19 pandemic, which may have influenced who was willing or able to participate or their ability to adhere to dietary instructions. This trial lacked a control group to which participants could have been randomized to, and thus, statements cannot be made about causality. Another limitation of our trial is that retention for outcomes varied from 47% for the final HbA1c level to 82% for the final body weight. However, this is consistent with other lifestyle interventions among young adults and suggests the need for additional retention strategies. Our results should be replicated in a larger, more diverse sample.

## Conclusions and future directions

Overall, the results of this pilot study suggest that a very low-carbohydrate eating pattern may be a feasible and acceptable approach for weight loss in some young women with obesity.

### Supplementary Information

Supplementary Material 1.

## Data Availability

The datasets generated during and/or analyzed during the current study are available from the corresponding author on reasonable request.
